# The Extra Virgin Olive Oil Polyphenol Oleocanthal Exerts Antifibrotic Effects in the Liver

**DOI:** 10.3389/fnut.2021.715183

**Published:** 2021-10-04

**Authors:** Daniela Gabbia, Sara Carpi, Samantha Sarcognato, Luana Cannella, Martina Colognesi, Michela Scaffidi, Beatrice Polini, Maria Digiacomo, Jasmine Esposito Salsano, Clementina Manera, Marco Macchia, Paola Nieri, Maria Carrara, Francesco Paolo Russo, Maria Guido, Sara De Martin

**Affiliations:** ^1^Department of Pharmaceutical and Pharmacological Sciences, University of Padova, Padova, Italy; ^2^Department of Pharmacy, University of Pisa, Pisa, Italy; ^3^NEST, Istituto Nanoscienze-CNR and Scuola Normale Superiore, Pisa, Italy; ^4^Department of Medicine, University of Padova, Padova, Italy; ^5^Interdepartmental Research Center “Nutraceuticals and Food for Health”, University of Pisa, Pisa, Italy; ^6^Doctoral School in Life Sciences, University of Siena, Siena, Italy; ^7^Department of Surgery, Oncology and Gastroenterology, University of Padova, Padova, Italy

**Keywords:** liver fibrosis, hepatic stellate (Ito) cell (HSC), oleocanthal, extra virgin olive oil (EVOO), oxidative stress, inflammation, miRNA

## Abstract

Liver fibrosis, which is the outcome of wound-healing response to chronic liver damage, represents an unmet clinical need. This study evaluated the anti-fibrotic and anti-inflammatory effects of the polyphenol oleocanthal (OC) extracted from extra virgin olive oil (EVOO) by an *in vitro/in vivo* approach. The hepatic cell lines LX2 and HepG2 were used as *in vitro* models. The mRNA expression of pro-fibrogenic markers, namely alpha-smooth muscle actin (α-SMA), collagen type I alpha 1 chain (COL1A1), a panel of metalloproteinases (MMP1, MMP2, MMP3, MMP7, MMP9) and vascular endothelial growth factor A (VEGFA) as well as the pro-oxidant genes NADPH oxidases (NOXs) 1 and 4 were evaluated in TGF-β activated LX2 cells by qRT-PCR. α-SMA and COL1A1 protein expression was assessed by immunofluorescence coupled to confocal microscopy. VEGFA release from LX2 was measured by ELISA. We also evaluated the amount of reactive oxygen species (ROS) produced by H_2_O_2_ activated- HepG2 cells. *In vivo*, OC was administered daily by oral gavage to Balb/C mice with CCl_4_-induced liver fibrosis. In this model, we measured the mRNA hepatic expression of the three pro-inflammatory interleukins (IL) IL6, IL17, IL23, chemokines such as C-C Motif Chemokine Ligand 2 (CCL2) and C-X-C Motif Chemokine Ligand 12 (CXCL12), and selected miRNAs (miR-181-5p, miR-221-3p, miR-29b-3p and miR-101b-3p) by qRT-PCR. We demonstrated that OC significantly downregulated the gene/protein expression of α-SMA, COL1A1, MMP2, MMP3, MMP7 and VEGF as well as the oxidative enzymes NOX1 and 4 in TGFβ1-activated LX2 cells, and reduced the production of ROS by HepG2. *In vivo* OC, beside causing a significant reduction of fibrosis at histological assessment, counteracted the CCl_4_-induced upregulation of pro-fibrotic and inflammatory genes. Moreover, OC upregulated the anti-fibrotic miRNAs (miR-29b-3p and miR-101b-3p) reduced in fibrotic mice, while downregulated the pro-fibrotic miRNAs (miR-221-3p and miR-181-5p), which were dramatically upregulated in fibrotic mice. In conclusion, OC exerts a promising antifibrotic effect *via* a combined reduction of oxidative stress and inflammation involving putative miRNAs, which in turn reduces hepatic stellate cells activation and liver fibrosis.

## Introduction

Extra virgin olive oil (EVOO), one of the main pillars of Mediterranean diet, displays numerous beneficial effects on human health, as for example the prevention of cardiovascular and related diseases, protection toward inflammatory bowel diseases, chemoprevention and reduction of neurological disorders and the incidence of neurodegeneration (1). EVOO contains 97–99% of lipids, mostly triglycerides, and 1–3% of minor components, accounted for exerting multiple antioxidant and bioactive properties. These minor components belong to different chemical classes, e.g., sterols, hydrocarbons (squalene, β-carotene), flavonoids, carotenoids, terpenoids, tocopherols, and polyphenols (hydroxytyrosol, oleuropein, oleocanthal) (2). For this peculiar composition, the European Food Safety Authority (EFSA) approved a health claim for virgin olive oils containing a minimum dose of 5 mg of naturally occurring polyphenols per 20 g of oil, i.e., “Olive oil polyphenols contribute to the protection of blood lipids from oxidative stress” (Commission Regulation (EU) 432/2012). Among EVOO polyphenols, oleocanthal (OC) is gaining attention due to its interesting biological activities. This secoiridoid was firstly discovered by Montedoro's group in the 90's (3) and named in 2005 by Beauchamp and his collaborators due to its pungent taste (4), which gives the typical strong prickling sensation in the throat of EVOO. Amongst the multiple beneficial effects of OC, its antioxidant and anti-inflammatory potential as well as its possible antitumoral effect on several cancers have recently been reviewed (5). Moreover, a recent study demonstrated that the supplementation with EVOO characterized by a high OC concentration had a positive effect on patients with metabolic syndrome and hepatic steatosis, by inducing a significant decrease of pro-inflammatory cytokines such as interleukin 6 (IL6), interleukin 17A (IL17A), tumor necrosis factor α (TNFα) and interleukin 1β (IL1β**)** (6), thus confirming the hepato-protective action of EVOO which had been observed in two previous human studies (7, 8). An experimental study of Al-Seeni and collaborators demonstrated that olive oil is able to protect the liver of rats from the toxicity induced by a 4-week carbon tetrachloride (CCl_4_) administration, improving all biochemical liver function tests and reducing inflammatory infiltration (9). CCl_4_ is an hepatotoxicant commonly used to obtain rodent models of liver fibrosis, because it is metabolized by the enzyme cytochrome P450 (CYP) 2E1 in highly reactive free radicals inducing oxidative stress and hepatic injury, leading to the trigger of fibrogenesis (10). Liver fibrosis represents the outcome of wound-healing response occurring in chronic liver diseases of different etiologies. Fibrosis can progress into liver cirrhosis which in turn may lead to liver failure and cancer (11). According to the Global Burden of Disease, chronic liver diseases are accounted as one of the major causes of mortality in the world, with more than 1.32 millions of deaths only in 2017 (12). The therapeutic options available for fibrotic patients are limited, and no specific anti-fibrotic medications have been approved, although many different candidate drugs are in the pipeline.

One of the main actors in the deposition of fibrotic tissue in the liver is represented by hepatic stellate cells (HSCs), which are physiologically devoted to store lipids, such for example vitamin A, but could turn from a quiescent to a proinflammatory and profibrotic myofibroblast-like phenotype after a prolonged hepatic injury. Once activated, HSC promotes the remodeling of the extracellular matrix (ECM) through the upregulation of some metalloproteinases (MMPs), particularly MMP3, MMP7 and MMP9 (13, 14). An increased production of reactive oxygen species (ROS) can lead directly to HSC activation and induce hepatocyte inflammation (15), which can further sustain the production of ECM proteins by activated HSCs and fibroblasts. Many studies have demonstrated that HSC activation could also be promoted by the release of proinflammatory chemokines and cytokines, such as IL6 and transforming growth factor-β (TGF-β) from Kupffer cells (KCs), the resident macrophages of the liver (16, 17). KCs also produce large amount of nitric oxide that reacts with ROS, producing free radicals that sustain HSC activation and exacerbate the production of ECM. In this contest, oxidative stress and fibrosis seem to be causative related to disease progression. Therefore, targeting redox homeostasis has been proposed as a useful strategy to stop fibrosis progression and even induce its remission in controlled clinical trials (16). This strategy could be extremely interesting considering the lack of effective therapeutic options in patients with advanced fibrosis and cirrhosis.

In the light of these considerations, the aim of this study was the investigation of the anti-fibrotic and anti-inflammatory effects of OC extracted from EVOO by an *in vitro/in vivo* approach. First, we assessed the OC effect *in vitro* on the activation of LX-2 (a cell line mimicking HSC) by measuring the expression of the profibrotic genes α-smooth muscle actin (αSMA) e collagen type I alpha 1 chain (COL1A1), some MMPs and Vascular Endothelial Growth Factor A (VEGFA), which are involved in ECM remodeling and fibrogenesis, and markers of oxidative stress. i.e., the NADPH oxidases (NOX) 1 and 4. Then, we evaluated the OC effect *in vivo* in a mouse model of CCl_4_-induced liver fibrosis, by measuring a panel of pro-inflammatory cytokines, and the expression of some microRNAs (miRNAs) known to be involved in fibrogenesis and inflammation.

## Materials and Methods

### Oleochantal Extraction

OC was extracted from EVOO as previously reported (18), with slight modifications (19). The purification of OC was performed using advanced automated flash purification (Isolera™Prime 3.2.2, Biotage®) as described for the purification of oleacein (20). In the first step of purification, Biotage SNAP Ultra cartridge (HP-Sphere^TM^ 25μm) was chosen as stationary phase. The mobile phase was a mixture of CHCl_3_ **(A)** and ethyl acetate **(B)** shown in Table 1. The flow rate was 25 mL/min and for each tube 15 mL was collected. Fractions 40–60 (about 60 mg) containing OC were subjected to further purification using a Biotage® SNAP Ultra C18 12 g cartridge (HP-Sphere™ C18 25μm) as stationary phase. The mobile phase was a mixture of H_2_O (A) and ACN (B) shown in Table 2. The flow rate was 10 mL/min and for each tube 5 mL was collected. Fractions 30-40 (about 15 mg) contain pure oleocanthal (purity >95%). Identification and purity of the extracted compound were based on ^1^H NMR, LC-MS, and HPLC analyses (21). Purified OC was dissolved in DMSO to obtain a stock solution of 50 mM, that was freshly diluted in complete medium for the *in vitro* evaluations.

**Table 1:** Mobile phase used for the first step of OC purification.

**Mobile Phase (B%)**	**Fractions**	**Volume (mL)**
0%	1–36	540
From 0% to 45%	37–180	2,160
45%	181–186	90
From 45% to 100%	187–192	90
100%	193–198	90

**Table 2:** Mobile phase used for the further OC purification.

**Mobile Phase (B%)**	**Fractions**	**Volume (mL)**
From 5% to 10%	1–10	51
From 10% to 100%	11–61	255
100%	62–75	68

### Cell Cultures and Cell Viability Assay

LX2 (human hepatic stellate cell line) and HepG2 (human hepatocellular carcinoma cells) were maintained in DMEM with L-glutamine (1%) and streptomycin/penicillin (1%) supplemented with 3% or 10% FBS, respectively. Cell lines were maintained in humified 5% CO_2_-enriched atmosphere at 37 °C. Cell viability was assessed by means of MTT assay (22), after either 6, 24, and 48 h of OC treatment.

### mRNA Expression Analisys by qRT-PCR and VEGFA Quantification by ELISA Assay in LX2 Cell Line

To perform mRNA expression analysis, LX2 cells (15^*^10^4^ cell/well) were seeded into 6-well plates. After 24 h, cells were treated with TGF-β1 (2 ng/mL) to induce LX2 activation (23) with or without 2 μM OC for 24 h. At the end of the treatment, cell supernatants were collected and stored at−80°C to perform VEGFA quantification by means of a commercially available ELISA kit (Catalog #: ELH-VEGF, RayBiotech, Peachtree Corners, GA, USA). The determination of VEGFA concentration was performed following the manufacturer's instructions (24). Total mRNA from cell culture was extracted with TRIzol and Direct-zol RNA MiniPrep (Zymo Research, Irvine, CA, USA). QuantiNova SYBR Green RT-PCR Kit (Germantown, MD, USA) was used to perform qRT-PCR on an Eco Illumina Real-Time PCR system (San Diego, CA, USA). Primer sequences used for human cell analysis are reported in Table 3. They have been designed by means of Primer-BLAST (NCBI, NIH) and obtained by Eurofins Genomics (Milan, Italy). The qRT-PCR reaction was performed in a total reaction volume of 10 μL, and the thermal program was carried out as follows: 15 min at 50 °C and 2 min at 95 °C for the reverse transcription and 40 cycles of 15 s at 95 °C and 60 s at 60°C for PCR reaction. All the samples were run in triplicate. To calculate the relative mRNA expression according to 2^−ΔΔCt^ method, the cycle threshold (Ct) values were determined, and GAPDH was used as housekeeping gene (25).

**Table 3:** Primers for qRT-PCR analysis performed on human cell lines.

**Target**	**Forward**	**Reverse**	**Ref Seq**
α-SMA	CCTTTGGCTTGGCTTGTCAG	CGGACAGGAATTGAAGCGGA	NM_001141945.2
COL1A1	ACGTCCTGGTGAAGTTGGT	CAGGGAAGCCTCTCTCTCCT	NM_000088.4
MMP1	CACAGCTTTCCTCCACTGCTGCT	GGCATGGTCCACATCTGCTCTTG	NM_002421.4
MMP2	ACCTGGATGCCGTCGTGGAC	TGTGGCAGCACCAGGGCA	NM_004530.6
MMP3	GTTCCGCCTGTCTCAAGATGA	GGGACAGGTTCCGTGGGTA	NM_002422.5
MMP7	AAACTCCCGCGTCATAGAAAT	CCCTAGACTGCTACCATCCG	NM_002423.5
MMP9	GTCATCCAGTTTGGTGTCGC	GGACCACAACTCGTCATCGT	NM_004994.3
VEGFa	ATGGCAGAAGGAGGAGGGCA	ATCGCATCAGGGGCACACAG	NM_001025366.3
NOX1	CTGGTTGTTTGGTTAGGGCTG	TTCAAGCAGAGAGCAGACGC	NM_007052.5
NOX4	AGATGTTGGGGCTAGGATTGTGT	AATCTCCTGGTTCTCCTGCTTG	NM_016931.5
GAPDH	ACATCAAGAAGGTGGTGAAGCA	GTCAAAGGTGGAGGAGTGGTT	NM_002046.7

### Evaluation of α-SMA and COL1A1 Protein Expression by Immunofluorescence Coupled to Confocal Microscopy

We evaluated the effect of OC on the expression of the two fibogeninc proteins α-SMA and COL1A1 in LX2 cells activated with TGF-β1 (2 ng/mL). LX2 cells were seeded in a 24-well plate with glass coverslips and, after 24 h, were treated with TGF-β1 either in absence or presence of 2 μM OC for 24 h. The effect of OC was evaluated using untreated and TGF-β1-treated cells as negative and positive controls, respectively. At the end of the treatment, cells were fixed in 4% paraformaldehyde and, after whasing and blocking of the unspecific binding sites, incubated first with a mouse monoclonal primary antibody directed against either α-SMA (Catalog #: A5691, 1:500 dilution, Sigma-Aldrich, Milan, Italy) or COL1A1 (Catalog #: sc-293182, 1:100 dilution, Santa Cruz Biotechnology, Dallas, TX, USA) for 2 hours, and then with an Alexa Fluor 568 anti-mouse secondary antibody (1:500 dilution, Jackson ImmunoResearch Europe Ltd., Ely, UK) for 1 h. After treatment with a 2 mg/mL RNAse solution (Sigma-Aldrich, Milan, Italy), cell nuclei were stained blue with DAPI (100 pg/mL, Life Technologies, Monza, Italy). The images of the immunostained cells were acquired by means of a confocal microscope Zeiss LSM 800, using a 63X magnification. The ImageJ software was used to quantify the intensity of the fluorescent signal (26).

### Measure of ROS Production by HepG2 Cells

Intracellular ROS production in HepG2 cells was assessed by means of H_2_DCF-DA (27), with minor modifications. Briefly, cells were seeded into 96-well black plate (8,000 cell/well) and after 24 h treated with 60 μM hydrogen peroxide (H_2_O_2_) (28) either in presence or absence of 2 μM OC. After 3 h, 10 μM H_2_DCF-DA was added and incubated for 30 min at 37 C. 2′,7′-dichlorofluorescein fluorescence intensity was measured by means of VictorNivo multiplate reader (excitation: 480 nm, emission: 530 nm) (Perkin Elmer) and normalized to that measured for control untreated cells.

### Animal Studies

**Figure 1:**
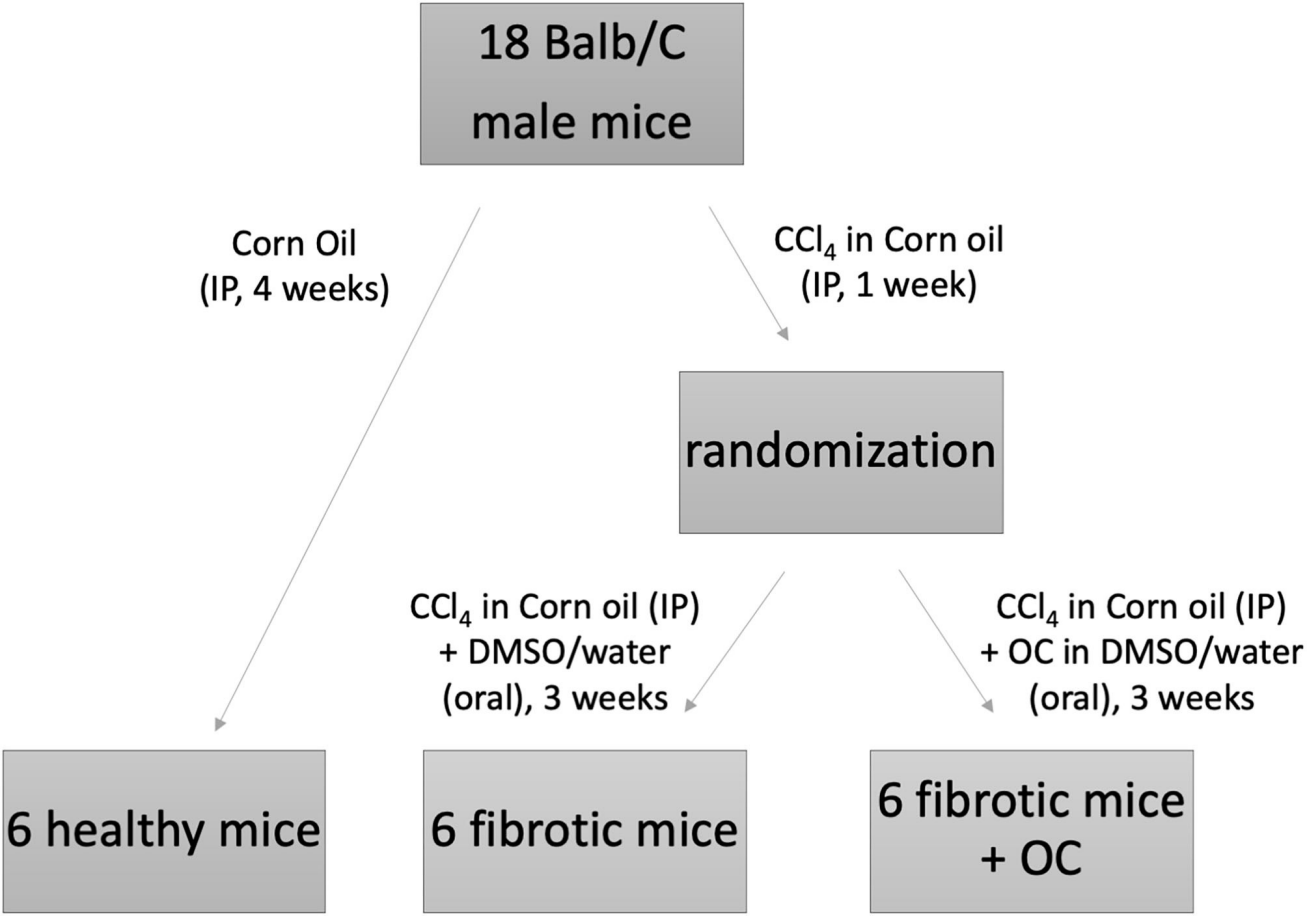
Experimental design of the *in vivo* study.

All animal studies were performed in compliance with national and European guidelines as described below, and suitable procedures were performed to minimize their pain and discomfort. Animals were kept in 4 or 5 per cage in the same room by the same personnel, with *ad libitum* access to the standard laboratory diet and tap water. They were monitored daily by the researchers involved in this study and/or by the personnel in charge for animal care (29). The experimental design, which has been approved by the Italian Ministry of Health (Auth. no. 201/2019-PR) is reported in Figure 1. To obtain the animal model of liver fibrosis, twelve Balb/C male mice (6 weeks old) were treated with CCl_4_ in corn oil (0.5 mL/Kg) by intraperitoneal (IP) injection three times a week for 4 weeks (30). After the first week of CCl_4_-treatment, the 12 mice were randomized into 2 experimental groups (*n* = 6 per group), one (“fibrotic mice”) treated with CCl_4_ IP and OC vehicle (oral gavage, 1:10 solution of DMSO:water); the other (“fibrotic mice + OC”) treated with both CCl_4_ and OC. OC was administered daily by oral gavage for 3 weeks (10 mg/Kg dose, 1:10 dilution with water from the 50 mM stock solution in DMSO), until the sacrifice. A control group of healthy mice (*n* = 6) was injected IP with the CCl_4_-vehicle (corn oil). At sacrifice, liver tissues were collected for further analysis; a portion was embedded in 4% neutral buffered formalin and the remaining tissue was nitrogen frozen and stored at −80°C until used. Liver tissue slides were stained with hematoxylin-eosin (H&E) and Masson's trichrome stain for fibrosis evaluation. The histological examinations of liver sections have been blindly performed by the same pathologist (15).

### Gene Expression Analysis on Liver Tissue by qRT-PCR

Total RNA was obtained from 150 mg of liver tissue after homogenization and purification with a commercial RNA isolation kit (Promega Corporation, Madison, WI, USA), following the manufacturer's instructions. To prevent possible genomic DNA contamination, a step with DNase treatment was carried out. Purified RNA was eluted RNase-free water, aliquoted and stored at −80 °C until use. To quantify the expression of the genes reported in Table 4, qRT-PCR was performed using a one-step SYBRgreen commercial kit (Takara, Mountain View, CA, USA), using β-actin as the housekeeping gene (31). Relative mRNA expression was calculated by the 2^−ΔΔCt^ method.

**Table 4:** Primers for qRT-PCR analysis on murine liver tissues.

**Target**	**Forward**	**Reverse**	**Ref Seq**
α-SMA	GCTACGAACTGCCTGACGG	GCTGTTATAGGTGGTTTCGTGGA	NM_007392.3
COL1A1	AGCACGTCTGGTTTGGAGAG	GACATTAGGCGCAGGAAGGT	NM_007742.4
IL6	CCGGAGAGGAGACTTCACAG	TCCACGATTTCCCAGAGAAC	NM_031168.2
IL10	GCTGGACAACATACTGCTAACC	CTGGGGCATCACTTCTACCA	NM_010548.2
IL23	GAGCAACTTCACACCTCCCT	CTGCCACTGCTGACTAGAAC	NM_031252.2
CXCL2	GGCTAACTGACCTGGAAAGGA	GGTACGATCCAGGCTTCCC	NM_009140.2
CXCL12	CTTTGTAACTCGCTCCTCCCT	GGGAAGAGTTTACCGTCAGGTT	NM_001012477.2
β-actin	ATGTGGATCAGCAAGCAGGA	AAGGGTGTAAAACGCAGCTCA	NM_007393.5

### Evaluation of Hepatic miRNAs Expression

Total miRNAs were extracted and purified from frozen hepatic tissue by using the miRNeasy Mini Kit (Qiagen, Hilden, Germany). Reverse transcription of the extracted miRNAs was performed by the miScript Reverse Transcription Kit (Qiagen, Hilden, Germany). cDNA was diluted 1:3 in RNase-free water and then qPCRs were performed in triplicate using the miScript SYBR-Green PCR kit (Qiagen, Germany) on the MiniOpticon CFX 48 real-time PCR Detection System (Bio-Rad, Hercules, CA, USA). MiScript Primer Assays specific for mmu-miR-181-5p (MIMAT0000210), mmu-miR-221-3p (MIMAT0000669), mmu-miR-29b-3p (MIMAT0000127), mmu-miR101b-3p (MIMAT0000616) and mmu-RNU6 were obtained from Qiagen, according to a validated protocol, set up and optimized by the authors (20). The expression of miRNAs was calculated by the 2^−ΔΔCt^ method, using RNU6 as the housekeeping gene.

### Statistical Analyses

Statistical analyses were performed using GraphPad Prism software ver. 8.0 (San Diego, CA, USA). One-way ANOVA followed by the the Tukey's *post-hoc* test was used to assess the eventual significant difference between groups. P < 0.05 was considered statistically significant. If not otherwise stated, data are presented as mean ± S.D of at least 3 independent experiments.

## Results

### *In vitro* Evaluation of the Antifibrotic Activiy of OC

#### Effect of OC on LX2 Activation

**Figure 2:**
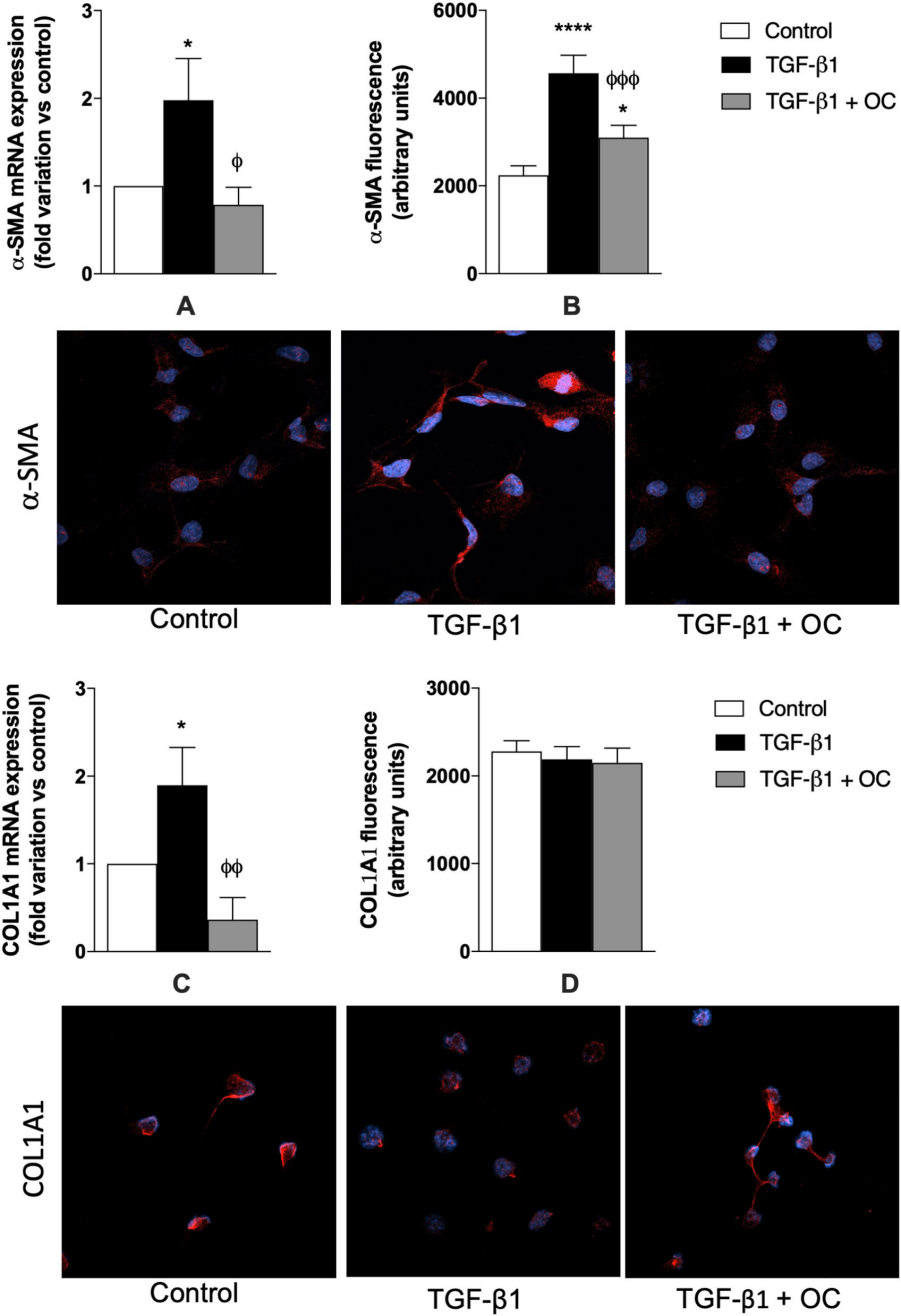
Effect of OC on α-SMA **(A, B)** and COL1A1 **(C, D)** mRNA and protein expression in LX2 cell line. *P < 0.05, ****P < 0.0001 *vs*. control, ^ϕ^*P* < 0.05, ^ϕϕ^*P* < 0.01, ^ϕ*ϕϕ*^*P* < 0.001 *vs*. TGF-β1 treated cells. Representative images of α-SMA and COL1A1-related red fluorescence are shown. Cell nuclei are stained blue with DAPI. At least 8 fields per coverslip have been analyzed to obtain the quantification of fluorescence. Results are the mean of 3 independent experiments and have been analyzed by one-way ANOVA followed by the Tukey's *post hoc* test.

In this study, LX2 cells were used as *in vitro* tools to assess the potential OC anti-fibrotic effect on fibrosis and HSC activation. First, the MTT assay was performed to exclude effects of OC on LX2 viability (Supplementary Figure 1). OC displayed a safe cytotoxicity profile on LX2 cells, since only at its highest concentration (25 μM) a slight and non-significant reduction in cell viability could be observed after 24 and 48 h of incubation (24% and 21%, respectively), whereas no effect at all was evident after a shorter incubation (6 h) of LX-2 cells at none of the tested OC concentrations. In the light of these results, the non-cytotoxic 2 μM OC was chosen for the following *in vitro* assays. To investigate the *in vitro* anti-fibrotic effects of OC, TGF-β1 was used as a prototypical stimulus to induce the activation of quiescent HSCs. As shown in Figure 2, TGF-β1 was able to upregulate significantly the expression of the two profibrotic genes α-SMA (Figure 2A) and COL1A1 (Figure 2C, *p* < 0.05 for both genes), and this increase was counteracted by OC treatment (*p* < 0.05 and *p* < 0.01 *vs*. TGF-β1-treated cells, respectively). As far as the protein expression is concerned, we obtained similar results for α-SMA, since the dramatic increase of its expression induced by TGF-β1 was counteracted by OC treatment, although basal levels could not be restored. On the contrary, no effect could be observed for the protein expression of COL1A1, which was similar in treated and untreated cells.

**Figure 3:**
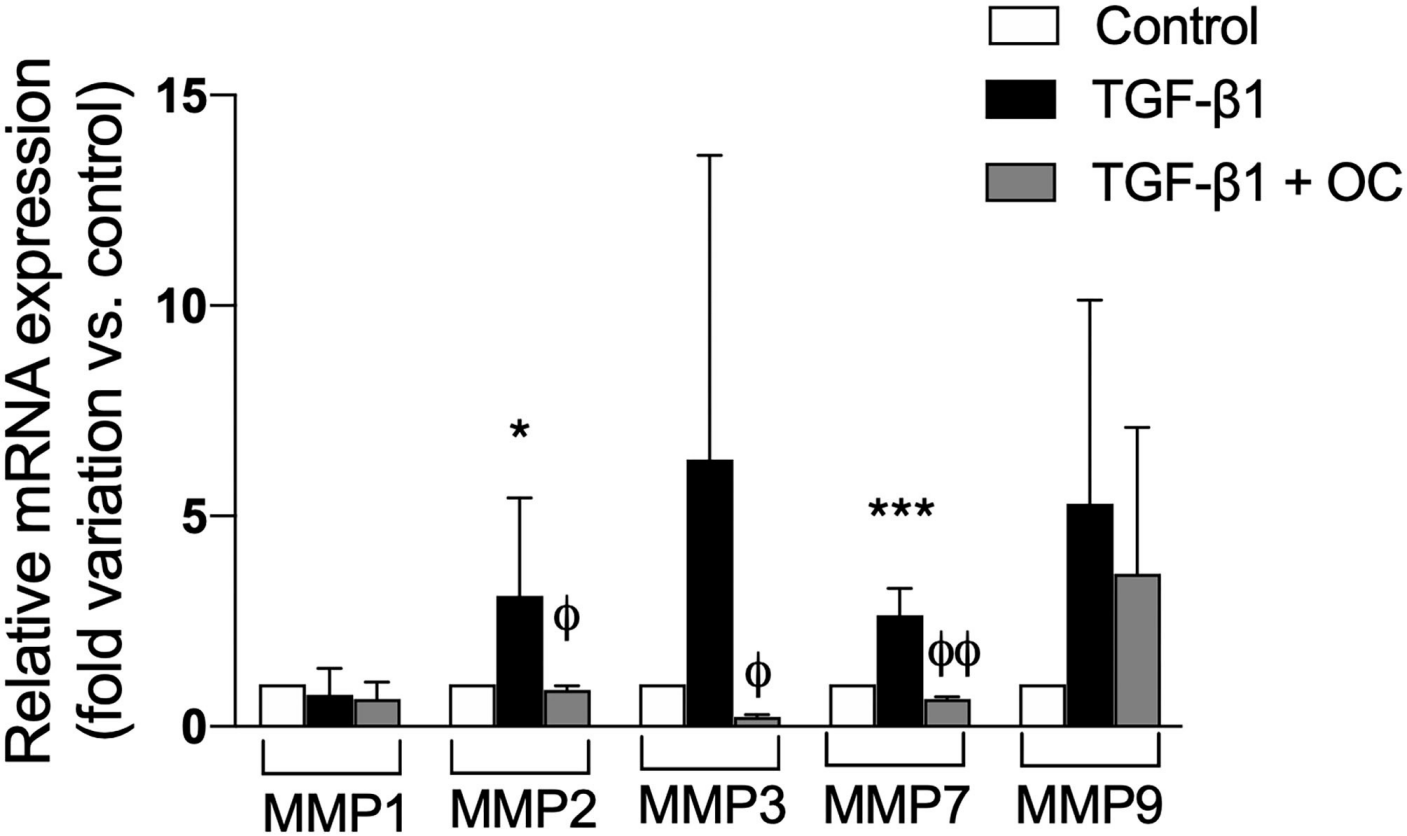
Effect of OC on MMPs gene expression in LX2 cell line. *P < 0.05, ***P < 0.001 *vs*. control, ^ϕ^*P* < 0.05, ^ϕϕ^*P* < 0.01 *vs*. TGF-β1 treated cells. Results are the mean of 3 independent experiments performed in triplicate and have been analyzed by one-way ANOVA followed by the Tukey's *post hoc* test.

Since another hallmark of liver fibrogenesis is the remodeling of ECM induced by the activation of HSCs, we investigated the effect of OC on the expression of some MMPs involved in this process. As shown in Figure 3, OC was able to counteract the mRNA expression increases of all the tested MMP isoforms induced by TGF-β1. In particular, the mRNA expression of MMP2, 3 and 7 was significantly reduced, whereas only a decreasing tendency could be observed for MMP9. MMP1 mRNA expression did not change.

**Figure 4:**
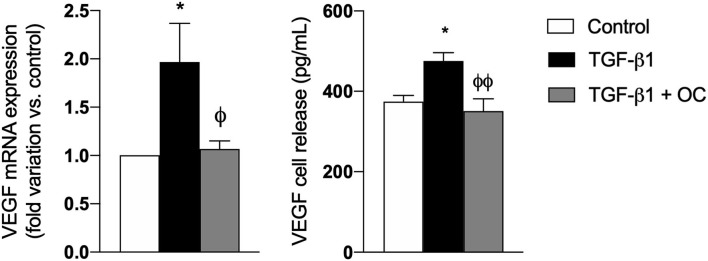
Effect of OC on VEGF mRNA expression **(A)** and release **(B)** from LX2 cell line. *P < 0.05 *vs*. control, ^ϕ^*P* < 0.05 *vs*. TGF-β1 treated cells. ^ϕϕ^*P* < 0.01 *vs*. TGF-β1 treated cells. Results are the mean of 3 independent experiments performed in triplicate (qRT-PCR) or in duplicate (ELISA) and have been analyzed by one-way ANOVA followed by the Tukey's *post hoc* test.

Furthermore, since it is well known that Vascular Endothelial Growth Factor-a (VEGFA) markedly increases during fibrogenesis and activates multiple downstream signaling pathways promoting the transition from liver fibrosis to HCC (32, 33), we investigated the effect of OC on its mRNA expression and release. As shown in Figure 4, OC significantly downregulated both gene expression and release of VEGFA from TGF-β1-activated LX2 cells, restoring their physiological levels.

**Figure 5:**
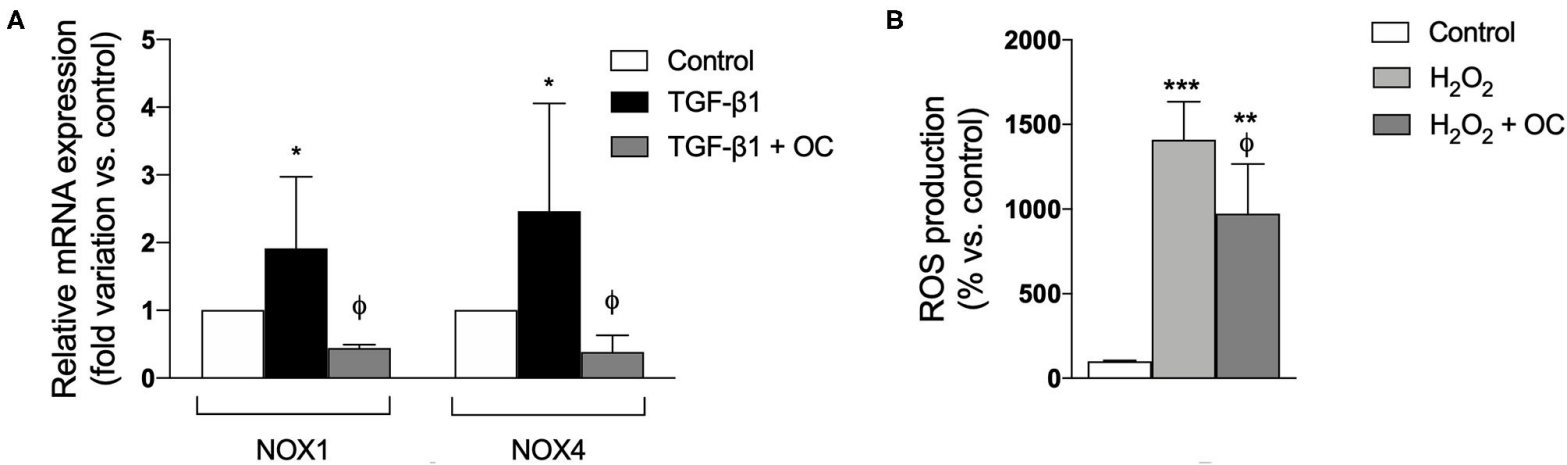
Effect of OC on gene expression of NOX1 and NOX4 in LX-2 cells **(A)** and ROS production by HepG2 cells treated with H_2_O_2_ **(B)**. *P < 0.05, **P < 0.01, ***P < 0.001 *vs*. control, ^ϕ^P < 0.05 *vs*. TGF-β1-treated cells **(A)** or *vs*. H_2_O_2_-treated cells **(B)**. Results are the mean of 3 independent experiments performed in triplicate and have been analyzed by one-way ANOVA followed by the Tukey's *post hoc* test.

To ascertain whether OC exerts antioxidant effects, we investigated its effects on oxidative stress by evaluating the mRNA expression of two isoforms (1 and 4) of NOXs, the main enzymes involved in hepatic ROS production. We demonstrated that OC significantly decreased NOX1 and NOX4 gene expression with respect to those observed in TGF-β1-activated LX2 cells (Figure 5A). Moreover, OC was also able to significantly reduce ROS production by the hepatocyte-like HepG2 cells stimulated by hydrogen peroxide (H_2_O_2_) (Figure 5B).

### *In vivo* OC Effects on a Murine Model of Liver Fibrosis

#### Effect of OC on Liver Fibrosis Markers

**Figure 6:**
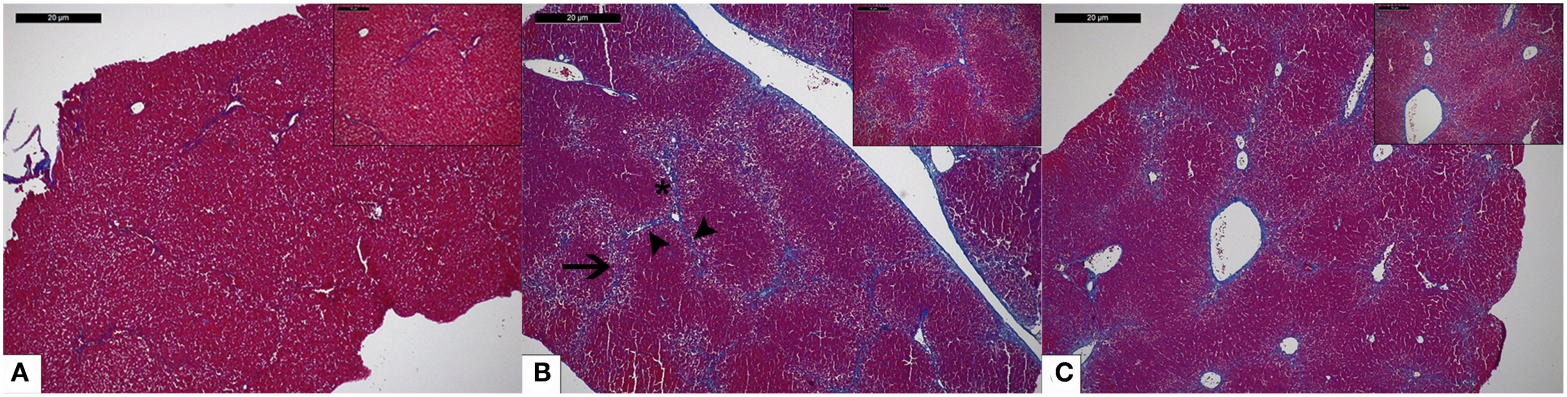
Representative photomicrographs of liver histology of mice included in the different study groups. **(A)** The liver of a healthy mouse from the control group shows no fibrosis (Masson's trichrome stain; original magnification 5x, upper right: original magnification 10x); **(B)** CCl_4_-induced liver fibrosis in a mouse from the untreated group. Central-central (asterisk), central-portal (arrowheads), and portal-portal (arrow) fibrous septa are shown (Masson's trichrome stain; original magnification 5x, upper right: original magnification 10x); **(C)** In mice treated with OC, liver histology shows regressing fibrosis (only focal thin incomplete fibrous septa are visible) (Masson's trichrome stain; original magnification 5x, upper right: original magnification 10x).

**Figure 7:**
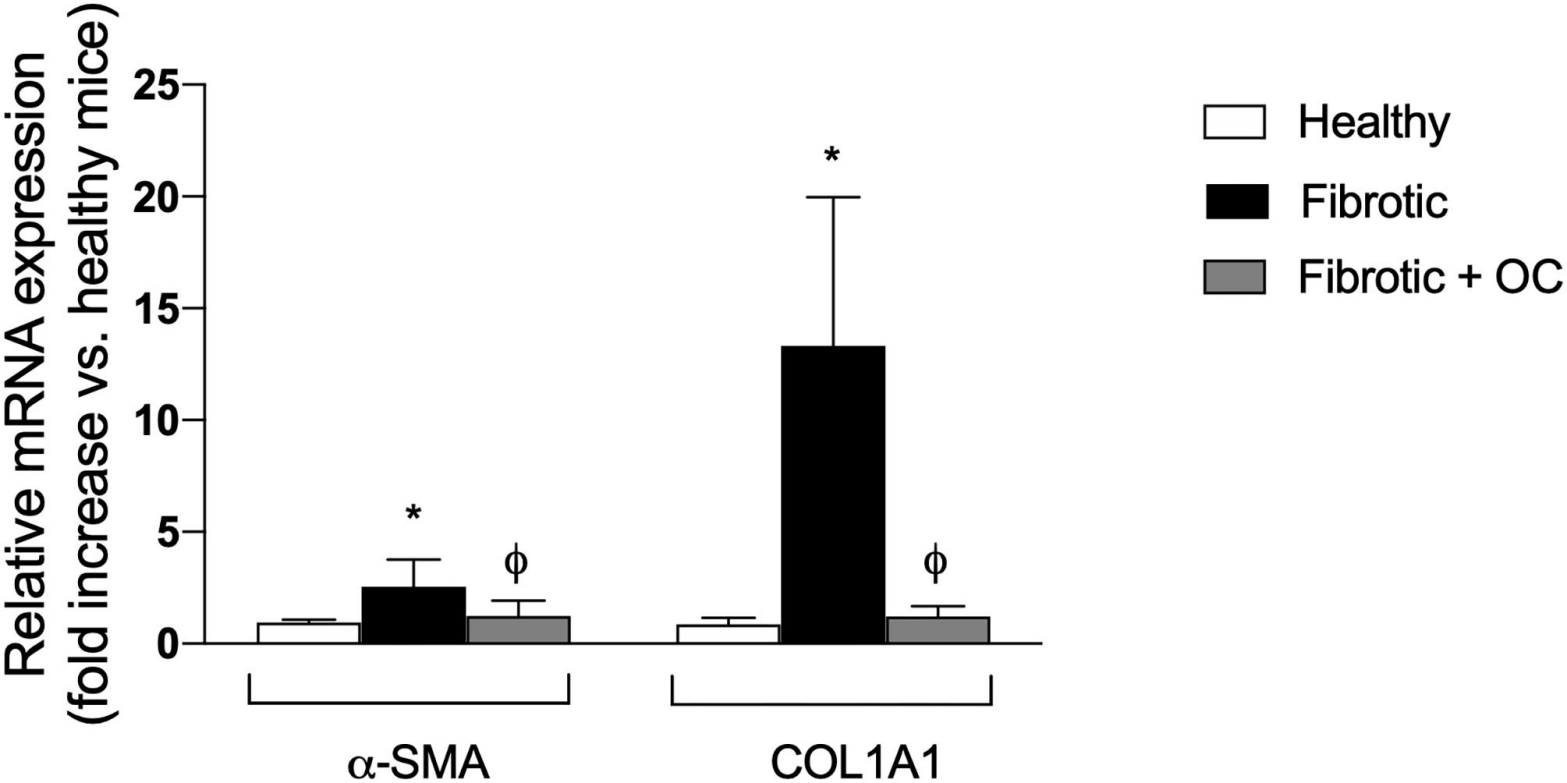
Effect of OC on the hepatic expression of the fibrogenic genes α-SMA and COL1A1 in the mouse model of fibrosis. *P < 0.05 *vs*. control, ^ϕ^ *P* < 0.05 *vs*. fibrotic mice. Results are obtained by analyzing 6 animals per group in triplicate and have been analyzed by one-way ANOVA followed by the Tukey's *post hoc* test.

As shown in Figure 6, livers of CCl_4_-treated animals showed widespread centri-lobular necro-inflammatory foci and bridging necrosis, with ceroid-containing histiocytes. This condition was slightly improved by OC treatment. In fibrotic animals, central to central (C-C), portal to central (P-C) and portal to portal (P-P) fibrous septa could be observed, whereas only isolate, thin, C-C septa were observed in OC treated animals. Accordingly, CCl_4_ treatment induced a marked increase of the mRNA expression of both α-SMA and COL1A1 with respect to healthy animals. This increase was counteracted by the administration of OC, which significantly downregulated α-SMA and COL1A1 expression, restoring their physiological levels (Figure 7).

#### Effect of OC on the Expression of miRNA Involved in Fibrogenesis

**Figure 8:**
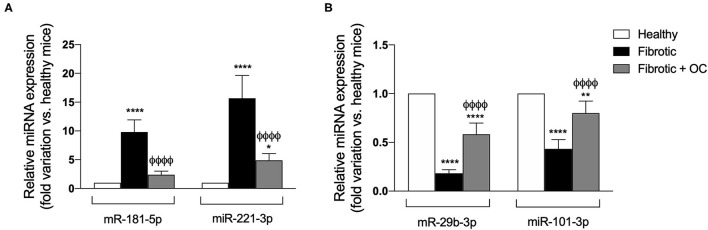
Effect of OC on the hepatic expression of pro-fibrotic **(A)** and anti-fibrotic **(B)** miRNAs in a mouse model of fibrosis. *P < 0.05, **P < 0.01, ****P < 0.0001 *vs*. control, ^ϕ*ϕϕϕ*^ *P* < 0.0001 *vs*. fibrotic mice. Results are obtained by analyzing 6 animals per group in triplicate and have been analyzed by one-way ANOVA followed by the Tukey's *post hoc* test.

As shown in Figure 8A, the hepatic expression of the profibrotic miR-181-5p and miR-221-3p resulted to be increased by CCl_4_ administration, as expected. Notably, OC decreased this upregulation significantly. Conversely, the two antifibrotic miRNAs miR-29b-3p and miR-101b-3p were downregulated in fibrotic mice, and OC counteracted this effect (Figure 8B).

#### Effect of OC on Hepatic Inflammation

**Figure 9:**
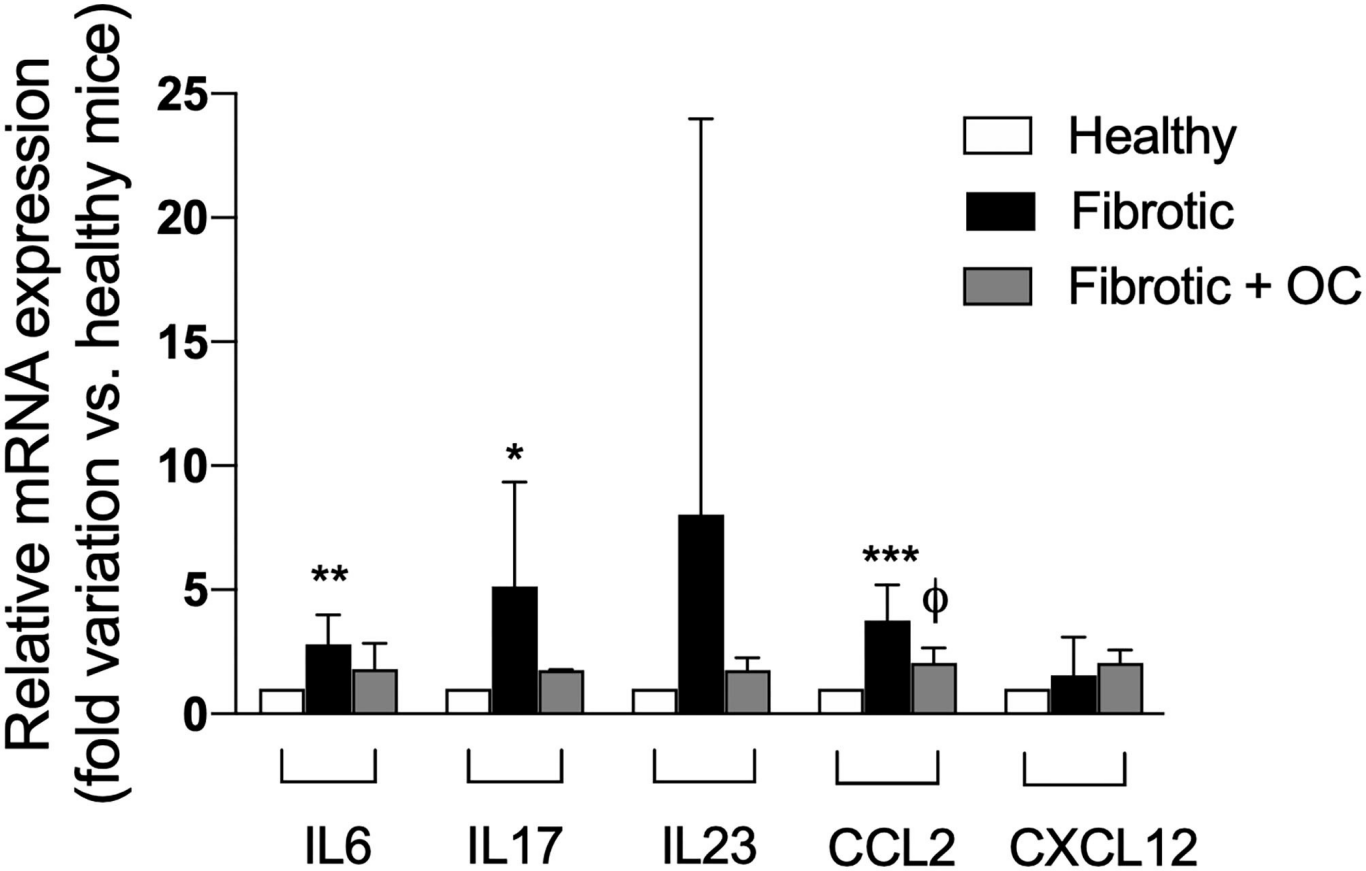
Effect of OC on hepatic gene expression of inflammatory cytokines in a mouse model of fibrosis. *P < 0.05, **P < 0.01 and ***P < 0.001 *vs*. control mice, ^ϕ^ *P* < 0.05 *vs*. fibrotic mice. Results are obtained by analyzing 6 animals per group in triplicate and have been analyzed by one-way ANOVA followed by the Tukey's *post hoc* test.

As reported in Figure 9, the administration of CCl_4_ induced an upregulation of the three tested pro-inflammatory ILs with respect to healthy animals, and this increase was significant for IL6 and IL17. OC decreased the pro-inflammatory IL mRNA expression to the levels of healthy animals, thus confirming anti-inflammatory effect of OC. Moreover, the significant CCL2 upregulation observed after CCL_4_-treatment was counteracted by the oral administration of OC, whereas the gene expression of CXCL12 was not affected either by CCl_4_ and/or OC in fibrotic mice.

## Discussion

In this study, we assessed the potential of the polyphenol OC extracted from EVOO toward hepatic fibrosis using an *in vitro/in vivo* approach to better describe the mechanism(s) of its effects. We demonstrated that OC exerted antifibrotic and anti-inflammatory effects and was also able to regulate the expression of a panel of miRNA involved in hepatic fibrogenesis. We focused on OC because the hepatoprotective properties of polyphenols or polyphenol-rich extracts obtained from several plants have been extensively demonstrated in numerous studies performed both *in vitro* and in different models of liver disease, including CCl_4_-induced liver injury (34, 35). Among these compounds, polyphenols extracted from EVOO have been investigated for their beneficial effects on liver health, which has been often linked to their antioxidant activity. Noteworthy, it has been demonstrated that EVOO containing high concentration of OC improved hepatic steatosis in patients with metabolic syndrome and reduced some pro-inflammatory cytokines (6).

Since one of the main players in hepatic fibrogenesis is the activation of quiescent HSCs that promote collagen deposition and ECM remodeling, we first investigated the effect of OC on a cellular model of activated HSC (TGF-β1-treated LX2 cells). OC significantly decreased the upregulation of α-SMA mRNA and protein caused by TGF-β1. On the contrary, COL1A1 protein expression did not change, probably because the modulation of COL1A1 protein expression may require a longer time in our experimental conditions than α-SMA. In fact, COL1A1 mRNA expression exactly reflects that of α-SMA, being upregulated by TGF-β1 and restored to normal levels by OC treatment. Since the upregulation of these two genes is the first step toward fibrogenesis, we conclude that *in vitro* OC seems to exert an antifibrotic effect by reducing HSC transition from a quiescent to a pro-fibrotic status. Moreover, it has been demonstrated that HSC activation promotes ECM remodeling and fibrotic tissue deposition through the modulation of some MMP isoforms (13, 14). Thus, we evaluated OC ability in affecting MMP mRNA expression in LX2 cells, observing that MMP2, 3 and 7 were significantly reduced by OC treatment, whereas only a decreasing tendency could be observed for MMP9, probably due to the high variability observed in the experiments. These results are in line with experimental and clinical observations, demonstrating the intriguing interplay between MMPs and collagen deposition, and reporting increased hepatic and plasmatic concentrations of some MMPs (in particular, MMP2, 7 and 9) in liver fibrosis (36, 37).

Another mechanism involved in HSC-mediated fibrogenesis is the release of pro-angiogenesis factors that induce both scar tissue proliferation and neo-angiogenesis, such as VEGFA (38, 39). In addition, it should be noticed that liver fibrosis can induce a hypoxic environment, which promotes the production of angiogenic factors, including VEGF and angiopoietin, and these factors lead to increased angiogenesis (40–42). We demonstrated that OC treatment decreased both mRNA expression and release of VEGFA from TGF-β1-activated LX2 cells, suggesting that its mechanism probably involves a complex modulation of HSC activation. Since oxidative stress plays a pivotal role in fibrogenesis and it has been demonstrated that OC displays antioxidant effect on various cell lines (5), we investigated its effect on oxidative stress-related pathways. Investigating oxidative stress is of particular relevance in fibrosis, since it well known that the pro-oxidative activity of the NOXs is increasing upon the activation of the receptor for advanced glycation endproducts (RAGE), caused by its interaction with ligands induced during inflammation (11). Beside NOXs, RAGE can in turn activate the inflammatory nuclear factor kappa-light-chain-enhancer of activated B cells (NF-κB) pathway, leading to a vicious cycle in which oxidative stress and inflammation generate a sustained pro-fibrotic response by reciprocal synergic actions (11, 43). In this context, we first observed that OC treatment downregulated the mRNA expression of two isoforms of NOXs i.e., NOX1 and NOX4, peculiar enzymes involved in hepatic ROS production, in TGF-β1-activated LX2 cells. Notably, these two NOX isoforms are significantly increased during the fibrotic process, and some NOX inhibitors are currently under preclinical and clinical evaluation as anti-fibrotic agents (44, 45). Furthermore, since the ROS produced by HSCs and other liver cells in the first stages of fibrogenesis can target hepatocytes contributing to their inflammation, and hepatocytes could in turn start to be active player in this process perpetuating and sustaining hepatic oxidative stress by producing ROS (44), we investigated whether OC could exert a direct antioxidant effect on the hepatocyte-like cells HepG2. We demonstrated that OC was able to reduce the increase of ROS production generated by incubating HepG2 with H_2_O_2_. Therefore, these results indicate that OC can exert an antioxidant effect in the liver by downregulating NOXs in HSC and reducing ROS production from hepatocytes. Taken together, the results of the *in vitro* evaluations indicate that OC reduced HSC activation, decreasing the expression of both α-SMA and COL1A, and of some MMPs involved in ECM remodeling. Moreover, OC decreases the VEGFA release from HSCs and reduces hepatic oxidative stress affecting NOX1 and 4 expression and ROS production.

Based on these *in vitro* results, we tested the antifibrotic effect of OC *in vivo* on a murine model of liver fibrosis obtained by CCl_4_ administration to Balb/C mice. This model is widely used to study anti-fibrotic agents and represents a suitable model for the aims of this study, since CCl_4_ metabolism produces free radicals that increase oxidative stress, leading to hepatic fibrotic injury (30). CCl_4_-treatment induces evident histological changes in the liver parenchyma, whereas OC ameliorates liver histological status and downregulates the expression of α-SMA and COL1A1. Notably, many studies, recently reviewed by Su and collaborators (46), have reported that some miRNAs could act to promote or prevent some pathways involved in liver fibrosis development, being involved in lipotoxicity, oxidative stress and metabolic inflammation. Here, we investigated the effect of OC administration on the hepatic expression of four miRNAs, miR-181-5p, miR-221-3p (profibrotic), miR-29b-3p and miR-101b-3p (antifibrotic). OC was able to significantly decrease the upregulation of miR-181-5p and miR-221-3p induced by CCl_4_, whereas the two miRNAs miR-29b-3p and miR-101b-3p that were downregulated in our murine model of fibrosis, were increased by OC treatment. It has been reported that miR-221-3p is upregulated in mouse models of liver fibrosis (47) and human fibrotic biopsies (48). Concordantly, its deletion in hepatocytes prevented fibrogenesis in two different mouse models of fibrosis, induced either by injecting mice with CCl_4_ for 8 weeks, or by feeding mice with 3,5-diethoxycarbonyl-1,4-dihydrocollidine (DDC)-containing diet for 4 weeks (49). Furthermore, miR-221-3p contributes to the activation of NF-κB, a master regulator of inflammation, while its inhibition induces the inactivation of the NF-κB pathway (50). Moreover, the increased levels of miR-29b-3p induced by OC are in line with their anti-fibrotic role. miR-29b-3p level was remarkably reduced in patients with liver fibrosis (51). Mechanistically, miR-29b-3p overexpression repressed TGF-β1-induced collagen I protein and α-SMA expression, probably through the direct bind to signal transducer and activator of transcription 3 (STAT3) (51). Additionally, according to the miRTarBase database of experimentally validated microRNA-target interactions (52), COL1A1 is a validated target of miR-29b-3p (MIRT003678). Thus, it appears that OC may directly reduce the pro-fibrotic α-SMA and COL1A1 genes expression by increasing miR-29B-3p levels. Finally, recent studies have shown the anti-fibrotic role of miR-101-3p, which has been reported to suppress the increased LX-2 viability and the accumulation of ECM induced by TGF-β1 (53, 54). Moreover, miR-101-3p administration significantly improved liver function, relieved hepatic parenchyma damage, and reversed liver fibrosis by decreasing the accumulation of ECM components in a mouse model (53). Finally, the role of miR-181-5p in the fibrotic process appears to be still contentious, since Teng and collaborators classified miR-181 as a pro-fibrotic miRNA (55), while Zhang and collaborators as anti-fibrotic (56). Our data showed an increased expression of miR-181-5p in the liver tissue of the mice treated with CCl_4_, in line with the data published by Teng and co-workers. Taken together, our data suggest that the modulation of miRNA expression profile observed in OC-treated fibrotic mice concurs to the improvement of liver fibrosis.

Hepatic fibrogenesis is also promoted by the release of pro-inflammatory cytokines, particularly IL6, and some chemokines, such as for example CCL2, from KCs and, as stated above, the interplay of oxidative stress and inflammation initiates ad sustains the fibrogenesis process (16). Thus, we evaluated liver expression of three ILs, i.e., IL6, IL17 and IL23, and two chemokines, CCL2 and CXCL12 that are upregulated by CCL_4_ administration. The treatment with OC was able to downregulate the mRNA expression of pro-inflammatory ILs, thus confirming the anti-inflammatory effect already demonstrated in other *in vitro* and *in vivo* models (4, 57–60). Moreover, the marked CCL2 upregulation observed after CCL_4_-treatment was significantly counteracted by the OC administration. This finding is of particular relevance, since it has recently been demonstrated that TGF-β1-activated LX2 cells increase CCL2 production thereby upregulating macrophage-specific markers (61). Moreover, since CCL2 and its receptor CCR2 control the infiltration of monocytes into the liver (62), we can hypothesize that OC interferes with the hepatic recruitment of these cells, to reduce inflammation. However, OC did not affect the mRNA expression of the chemokine CXCL12, which directs neutrophil migration toward damage foci (63). However, its mRNA expression did not change in untreated fibrotic mice. Taken together, these results point that OC may act on hepatic resident or recruited macrophages, either by reducing monocyte infiltration or by modulating the interplay between these cells and HSCs, or both.

In conclusion, with this work we provide *in vitro* and *in vivo* evidence of the anti-fibrotic effect of the polyphenol OC extracted from EVOO. Furthermore, we linked its effects to a combined reduction of oxidative stress and inflammation involving putative miRNAs, which in turn leads to a decrease in HSC activation and a reduction of hepatic fibrosis. Although further studies are needed to elucidate mechanistic details and corroborate the translational value of these findings, our results help in gaining new insight into the beneficial effects of flavonoids contained in EVOO in a condition, such as hepatic fibrosis, for which therapeutic options are so far limited both in terms of number and efficacy.

## Data Availability Statement

The original contributions presented in the study are included in the article/Supplementary Files, further inquiries can be directed to the corresponding authors.

## Ethics Statement

The animal study was reviewed and approved by Italian Ministry of Health.

## Author Contributions

DG and SD: conceptualization. MG, SC, MM, and FR: methodology. DG, SC, SS, LC, MCo, MS, BP, MD, JE, and CM: investigation. MCa, SD, and PN: resources. DG and SC: data curation. DG, SC, and SD: writing original draft. SD, MG, and MD: review and editing. MG, PN, MM, and SD: supervision. DG: project administration. SD: funding acquisition. All authors listed have made a substantial, direct and intellectual contribution to the work, and approved it for publication.

## Funding

This research was supported by the University of Padova, grant code DEMA_SID19_01.

## Conflict of Interest

The authors declare that the research was conducted in the absence of any commercial or financial relationships that could be construed as a potential conflict of interest.

## Publisher's Note

All claims expressed in this article are solely those of the authors and do not necessarily represent those of their affiliated organizations, or those of the publisher, the editors and the reviewers. Any product that may be evaluated in this article, or claim that may be made by its manufacturer, is not guaranteed or endorsed by the publisher.
