# Supplementary material for: The Extra Virgin Olive Oil Polyphenol Oleocanthal Exerts Antifibrotic Effects in the Liver
Source: Front Nutr. 2021 Oct 4;8:715183. doi: 10.3389/fnut.2021.715183 (PMC8521071; doi:10.3389/fnut.2021.715183)
Supplement: Supplementary file 1 [file Data_Sheet_1.docx]

Supplementary Material


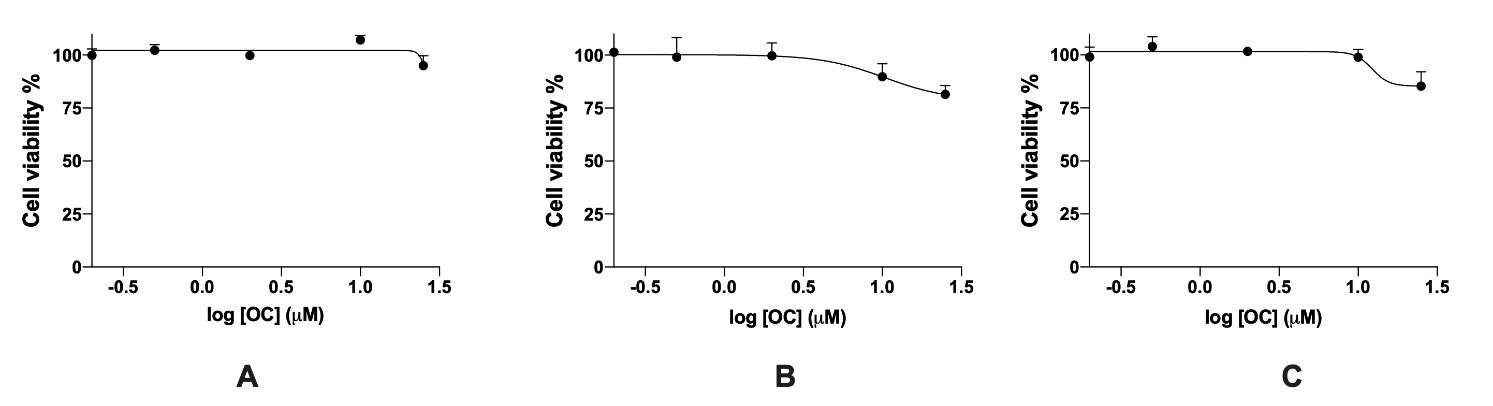


**Figure S1.** Effect of increasing concentrations of OC on LX2 cell viability after 6 (A), 24 (B) and 48 (C) hours of incubation assessed by means of MTT assay. Data are presented as mean ± SEM of 3 independent experiments performed in quadruplicate.
